# Immediate Open Access: The Good, the Bad, and the Impact on Academic Society Publishing

**DOI:** 10.1002/acr2.11547

**Published:** 2023-05-12

**Authors:** Amr H. Sawalha, Daniel H. Solomon, Kelli D. Allen, Patricia Katz, Ed Yelin

**Affiliations:** ^1^ University of Pittsburgh Pittsburgh Pennsylvania; ^2^ Brigham and Women's Hospital, Harvard Medical School Boston Massachusetts; ^3^ University of North Carolina & Durham VA Healthcare System Durham North Carolina; ^4^ University of California San Francisco San Francisco California

*We believe it is important to clarify that we are closely involved with journal publications of the American College of Rheumatology (ACR), as either Editors‐in‐Chief or Chair of the ACR's Committee on Journal Publications. Our involvement with the ACR journals comes with inherent conflicts of interest with the issue discussed. However, our involvement also positions us to anticipate what we foresee as potential unintended negative effects on academic publishing of the policy discussed herein. The dire consequences of this policy, if it takes effect without addressing the issues described below, necessitate expressing our opinion in a timely manner. Therefore, we provide our opinion without seeking approval of the Board of Directors of the ACR. The ACR continues to monitor and engage with stakeholders and policymakers around this policy change*.

Scientific discoveries are meaningless if not disseminated. Indeed, for new knowledge to have impact on how we understand or treat a disease, 3 components are paramount: scientific rigor, reproducibility, and timely dissemination.

In 2013, the White House Office of Science and Technology Policy (OSTP) recommended that articles based on research using public funds must be deposited into the public domain upon journal acceptance. The policy allowed for up to a 12‐month embargo before articles are made publicly available (1). Since articles were not immediately given Open Access status, the embargo helped publishers to sell subscriptions and recoup publication expenses. The dominant model remained “pay to read,” i.e., access via subscriptions, thereby providing a reasonable balance between public availability of federally funded publications and maintaining a healthy and rigorous scientific publication system. In fact, since 2008, the National Institutes of Health (NIH) has required submission of NIH‐funded, peer‐reviewed work to PubMed Central (2).

In recent years, Open Access scientific publishing has expanded, allowing authors the option to pay a fee and publish their work with immediate free access to the reader. These fees range from (USD) $3,000 to $10,000, depending on the journal. Open Access comes in several forms, including “Hybrid” and "Gold” Open Access. The American College of Rheumatology (ACR) journals *Arthritis & Rheumatology* and *Arthritis Care & Research* are both Hybrid journals, allowing authors to opt for Open Access or opt out. The ACR journal *ACR Open Rheumatology* is a Gold Open Access journal, requiring authors to pay a fee for immediate reader access. In the Hybrid model, publishers generate income from both the journal subscription fees and Open Access fees.

On August 25, 2022, the OSTP laid out an updated policy set to be in place by the end of December 2025, requiring immediate public access to articles generated from federally funded research. This policy would eliminate the allowable 12‐month embargo period. The OSTP mandate requires federal agencies to come up with plans to implement this new policy (3).

There are clear advantages to this new OSTP policy. New knowledge described in scientific manuscripts will be made freely available at the time of publication, providing researchers and scientists immediate access to published work in their fields, even without a subscription. This allows replication efforts and application of new medical knowledge in research and clinical practice at a faster pace. However, we believe that this new OSTP policy requires additional scrutiny, as it might not quite achieve the intended goals. Depending on how federal agencies apply the OSTP policy, we worry about the potential for unintended harmful effects.

The economic reality is that publishers will have to recoup their expenses (and make at least some financial profit) to survive, as in any business. If a 12‐month embargo is removed and published articles made immediately available, journal subscription revenue will almost certainly dwindle, and publishers will be forced to move toward a Gold Open Access model. Publishing an article in an ACR journal with immediate Open Access is currently associated with a publication fee ranging from (USD) $3,080 to $4,940, which the authors are expected to pay. Thus, the updated OSTP policy could potentially push the financial burden of making publications immediately available to the public onto the authors. The policy, in its current form, does not suggest or create resources for these additional publication fees, and the NIH has not clarified whether they will pay these fees. In a practical sense, authors will be forced to use research budgets to fund this new mandate.

The OSTP policy has the potential to increase inequity in science. Scientists will be forced into a pay‐to‐publish model. For some researchers with substantial funds, this will be manageable; however, many researchers will find these fees prohibitive. When funds are not available, publishing completed work might be delayed, hindering the dissemination of new knowledge. This potential outcome is exactly the opposite of the desired OSTP policy goal. Moreover, junior scientists, who often have limited funds, will be impacted more than established senior scientists. Researchers from countries with more limited resources will not have a chance to publish in prestigious journals that were forced by the new policy to switch from a subscription to a Gold Open Access model.

From the perspective of the publisher, an expanded pay‐to‐publish model will only be sustainable by increasing the volume of accepted manuscripts. This will likely negatively impact rigor and reproducibility in scientific publications, and further burden an already shrinking reviewer pool. We are already seeing a plethora of predatory journals, and the new policy will accelerate the move to low‐quality scientific publications; this is, again, exactly the opposite of the intended OSTP policy goal.

Publications from the ACR and other medical and scientific societies provide an important platform to publish the most significant advances in specific medical and scientific fields. Historically, some of the most impactful and paradigm‐shifting work has been published in society journals, and external scientific peer review is a key component. Encouraging a pay‐to‐publish model puts society journals (and medical societies) at substantial financial risk.

We strongly support public and immediate access to medical and scientific advances. However, we do not believe the new mandate released by OSTP addresses the likely negative impacts we foresee. We urge a more careful examination of the updated policy, a more extended time to hear concerns from medical societies and the public, and consideration of alternatives that can increase access to scientific publications while maintaining quality.

## AUTHOR CONTRIBUTIONS

All authors were involved in drafting the article or revising it critically for important intellectual content, and all authors approved the final version to be published.
